# Supplementary material for: Wearing N95 masks decreases the odor discrimination ability of healthcare workers: a self-controlled before-after study
Source: PeerJ. 2023 Mar 14;11:e14979. doi: 10.7717/peerj.14979 (PMC10022507; doi:10.7717/peerj.14979)
Supplement: Supplemental Information 2 [file peerj-11-14979-s002.docx]

### **Participant recruitment**

Posters were used to recruit participants at a large hospital in Wuhan. All the participants were healthcare workers older than 18 years old and less than 65 years old. The exclusion criteria were as follows: ① significant intellectual impairment; ② dependency on cigarettes; ③ a history of nasal or brain trauma; ④ the use of drugs may affect the olfactory sensation; ⑤ anosmia; and ⑥allergy to essence or paraffin wax. Pre-test questionnaires were distributed to each participant to obtain the following information: sex, age and history of rhinitis, nasal trauma or operation, smoking, anosmia, and influenza in the last 2 weeks.

### **Outcome measurement**

A 12-item odor discrimination ability test box produced by Jiangsu Kinsenheimer Biotechnology Co., Ltd. was used to test the participants’ olfactory discrimination ability. The product contains 12 wax blocks and answer cards, which can be used to score the olfactory function of the participants and indicate whether their olfactory discrimination ability has decreased. The score was noted as 1 when the participant could correctly identify the odor; in contrast, the score was noted as 0 when the participant could not. The highest possible score is 12, and the lowest score is 0. An olfactory score lower than 8 implies an impairment of an individual’s olfactory discrimination ability. This test has been approved to be reliable and mainly used in the diagnostic of Parkinson’s disease^6,7^.

For factors affecting odor recognition, participants were represented by "1" if they had such conditions as rhinitis or operation, and "0" if they did not.

We changed the names of the participants into numbers, but it should be noted that the same numbers in the column of each participant in the schedule do not represent the same participant, and their order is out of order. For example, the participant numbered 10 in backup 1 and the participant numbered 10 in backup 2 May not be the same person.

### **Methods**

After completing the pre-test questionnaire, each participant was asked to enter one room that had good ventilation. The 12-item odor discrimination test was administered to each participant when wearing an N95 mask (3M^™^ N95 respirator, catalogue number 1860), surgical mask (Winner^®^, Executive Standard: YY 0469-2011) and no mask in turn. Each wax block was presented for approximately 3 s and was held 2-3 cm away from the nostrils. There was an interval of 10 s between each block.

### **Sample size calculation**

To detect an important difference of 1 in the olfactory test score between the tests performed while wearing an N95 mask and a surgical mask, with a power of 0.9, and type I error of 0.05, the number of participants needed was 57. The sample size was increased to 72 to account for dropouts. In total, 141 participants were asked to complete the pre-test questionnaire. Among them, 3 did not complete the questionnaire. Seventy-one participants were excluded based on the exclusion criteria. It is worth noting that 61 of them were excluded due to dependency on cigarettes. Ten participants were excluded due to nasal trauma and anosmia. Finally, we included 67 participants in our study. The flowchart is shown in Figure 1.

### **Statistical methods**

In this study, nonparametric continuous variables were analyzed with the Wilcoxon paired test. The parametric continuous variables were analyzed with Student’s t-tests. Categorical variables were compared using chi-squared tests. A two-tailed P-value <0.05 was considered statistically significant. Statistical analysis of the data was performed with R 4.0.2.
